# Ceramic TiO_2_ Membrane Modification by Coal Fly Ash (CFA) Particles

**DOI:** 10.3390/membranes16050157

**Published:** 2026-04-29

**Authors:** Saidulla Faizullayev, Akbota Adilbekova, Joanna Kujawa, Wojciech Kujawski

**Affiliations:** 1 Faculty of Chemistry and Chemical Technology, Al-Farabi Kazakh National University, Almaty 050040, Kazakhstan; 2 Faculty of Chemistry, Nicolaus Copernicus University in Torun, 87-100 Torun, Poland

**Keywords:** titania membrane, coal fly ash, oil-in-water emulsion, membrane separation, membrane modification

## Abstract

Commercial TiO_2_ ceramic membranes were modified using a slip-casting method with coal fly ash (CFA) obtained from a thermal power plant, Almaty, Kazakhstan. The aim was to enhance membrane surface properties for improved oil-in-water emulsion separation while maintaining structural integrity. Suspension of CFA, stabilized with N-dodecylpyridinium chloride (DPC) and polyvinyl alcohol (PVA), was applied as a coating layer on the TiO_2_ surface and subsequently sintered under controlled conditions. The resulting membranes were characterized by SEM-EDX (scanning electron microscopy with energy-dispersive X-ray), Raman spectroscopy, contact angle measurements, and zeta potential analysis. The modified membranes exhibited increased hydrophilicity, as indicated by a reduction in water contact angle (WCA) from 43.6 ± 2° to approximately 0°, and a decrease in the underoil contact angle of water (UOCA) from 147.6 ± 2° to 87 ± 2°. Raman spectroscopy confirmed that the TiO_2_ structure remained predominantly rutile, with no additional crystalline phases detected from CFA. Despite the improved wettability, pure water and oil-in-water emulsion fluxes decreased slightly, while filtrates displayed smaller oil droplet sizes, indicating enhanced emulsion stability after passage through the modified surface. These findings demonstrate that CFA-modified TiO_2_ membranes can serve as a sustainable and cost-effective approach for treating emulsified wastewater, utilizing industrial waste to improve performance without compromising mechanical robustness.

## 1. Introduction

Crude oil and water emulsions produced during extraction and transportation are arising issue and require complex separation procedures [1,2]. There are various methods of breaking oil-in-water emulsions, including the most common one, which is thermochemical treatment. This method allows the selection of optimal demulsifiers and conditions (such as concentration of a demulsifying agent or treatment temperature) for almost any type of crude oil emulsion. However, a significant limitation of this method is the impossibility of developing a universally applicable demulsifier and standardized separation conditions because of differences in crude oil nature depending on the oilfields.

Among the promising approaches for oil-water separation is the use of membranes. Membranes are typically categorized into inorganic, organic membranes, or their mixed matrix, including different types of ceramic and polymeric membranes. However, ceramic membranes are often preferred owing to their chemical, thermal, and mechanical stability compared to polymeric membranes, which may undergo swelling, structural changes under harsh operating conditions, such as exposure to aggressive solvents, or high temperatures [3]. Eventually, swelling reduces porosity and flux significantly [4]. In addition, compared to polymeric membranes ceramic ones can be cleaned by burning out organic substances.

Membrane modification has been extensively studied to improve the performance and selectivity of membranes in various separation processes. One commonly used technique for membrane modification is incorporating functional materials onto the membrane surface. Among the various functional materials, titanium oxide (TiO_2_) has gained significant attention owing to its unique physicochemical properties, including high thermal stability, mechanical strength, and chemical resistance [5]. Depending on the grade and type, TiO_2_ also has a high surface area and photocatalytic activity, making it an ideal material for use in the fields of water treatment, gas separation, and energy conversion [6,7].

Various substances have been used to modify TiO_2_ membranes, including polymers, nanoparticles, and organic compounds. These modifications have been shown to alter the surface properties of the TiO_2_ membrane, leading to changes in permeability, selectivity, and fouling resistance. Likewise, fly ash is a promising material as a low-cost support and active material in production of sorbents, photocatalysts, and membranes [8,9]. Yusuff et al. [10] have reported that coal fly ash was employed as a low-cost support rather than as the primary active photocatalyst material in a ZnO-promoted TiO_2_ composite, and that the presence of trace metal oxides within the fly ash contributed beneficially to the photocatalytic activity by facilitating charge transfer and synergistic interactions.

In this study, commercial TiO_2_ membranes were modified by a slip-casting method using coal fly ash (CFA) provided by the local thermal power plant (TPP) in Almaty (Kazakhstan). CFA powder was selected for membrane modification as an accessible and cheap material which is a waste from coal burning at TPP. The choice of CFA is supported by its unique physicochemical properties. CFA is predominantly composed of aluminum and silicon oxides which contribute to the development of negatively charged surface when mixed with water. Such an approach is in line with the sustainability goal [11]. The application of CFA as a low-cost modifier for TiO_2_ membranes represents both an environmentally and functionally attractive approach.

The overarching aim of the presented work was to modify ceramic TiO_2_ membrane with the industrial waste, i.e., coal fly ash, and to characterize the materials systematically. The membrane performance has been compared to the pristine sample. Various spectroscopic (Raman spectroscopy) and microscopic (scanning electron microscopy with energy-dispersive X-ray (SEM-EDX) techniques, as well as surface charge and goniometric studies, have been implemented during the material and physicochemical analyses. Additionally, pure water flux measurements were conducted, and a synthetic oil-in-water emulsion was filtered through both modified and pristine membranes under varying pressure conditions.

## 2. Materials and Methods

### 2.1. Materials

(1-Hexadecyl)trimethylammonium bromide (98%) (HDTA) was purchased from Alfa Aesar (Karlsruhe, Germany). 1-Dodecylpyridinium chloride (DPC), Tween 80 (Polysorbate 80), and Heptane 95% were provided by Sigma Aldrich (Taufkirchen, Germany). Toluene 99.5% and polyvinyl alcohol (PVA) of MW = 72,000 kDa were bought from Avantor Performance Materials (Gliwice, Poland). Titanium oxide membranes with pore sizes of 1.4 µm and 47 mm in diameter were purchased from Tami Industries (Nyons, France). Coal fly ash (CFA) samples were obtained from the thermal power plant in Almaty (Kazakhstan), dried at 105 °C for 2 h and sieved by vibratory sieve shaker Analysette 3 (FRITSCH GmbH, Idar-Oberstein, Germany) with the smallest mesh size of 63 μm. The sieved fraction of CFA was then milled in a planetary ball mill SQM-0.4L (Changsha Samy Instrument & Equipment Co., Ltd., Changsha, China) at 400 rpm for 10 min.

### 2.2. Modification of Titania Ceramic Membrane

Membranes were modified utilizing a slip-casting method. In particular, the pristine TiO_2_ membrane was coated with a thin layer of a casting solution (Table 1). The casting solution was prepared by adding CFA to the deionized water, which was sonicated for 20 min. Then, 1-dodecylpyridinium chloride (DPC) was added followed by 20 min of sonication. Finally, PVA solution (12%) was added, and the mixture was homogenized for 24 h using a magnetic stirrer (at 45 °C and 570 rpm). Subsequently, a casting solution was poured onto the surface of the pristine TiO_2_ membrane and left for a few minutes. Then, the membrane was treated thermally following the sintering scheme proposed by Jedidi et al. [12]. The sintering program started from room temperature, with heating at 1 °C/min to 250 °C, where it was held for 1 h. Then, the temperature was increased at 2 °C/min to 800 °C and held for 2 h.

Preliminary SEM observations of membranes coated with a single deposition cycle revealed incomplete and non-uniform surface coverage, with only a thin and discontinuous layer of CFA particles. Therefore, multiple coating cycles were applied to ensure the formation of a more homogeneous and continuous surface layer. In this study, the coating procedure was repeated four times on the same membrane sample.

To ensure reproducibility, all modification steps were performed under controlled conditions, and the characterization measurements were conducted in replicate, showing consistent trends across experiments.

### 2.3. Preparation of Model Emulsion

The model oil-in-water emulsion was prepared by mixing 0.5 wt% of Tween 80 aqueous solution with 5 vol% of heptol (heptane and toluene in a ratio 7:3) mixture followed by homogenization for 5 min using homogenizer MPW-302 (Mechanika Precyzyjna, Warsaw, Poland). Tween 80 was chosen as an emulsifier because of its relatively high hydrophilic–lipophilic balance (HLB) value of 15, making it suitable to form oil-in-water emulsions. Moreover, Tween 80 is nonionic and known as a green emulsifier [13].

### 2.4. Materials Characterization

SEM-EDX images were obtained using Phenom ProX microscope (Thermo Fisher Scientific, Eindhoven, The Netherlands) with a field of view of 368 µm and 15 kV mode. Zeta potential measurements and particle size analysis were carried out using Litesizer 500, Anton Paar DLS-ELS analyzer (Anton Paar Gmbh, Graz, Austria). Particle size distribution and zeta potential measurements of coal fly ash were tested in the aqueous suspension, while zeta potential of the membranes was measured in inert electrolyte solutions across a pH range from 2.5 to 11. An electrokinetic analyzer (SurPass3, Anton Paar) (Anton Paar Gmbh, Graz, Austria) was used to perform the zeta potential titration as a function of pH. The surface zeta potential was determined in a 0.001 M KCl electrolyte solution, with pH adjusted using a 0.05 M HCl or 0.05 M NaOH solution, added automatically by the instrument’s titration unit. Water contact angle (WCA) values were measured to investigate how surface properties altered after the membrane modification using goniometer (Biolin Scientific, Gothenburge, Sweden). The sessile drop technique was utilized to measure WCA values of DI water and cyclohexane, while the captive bubble method was used to measure the underwater contact angle of air, water, and cyclohexane. Underwater (UWCA) and underoil (UOCA) contact angle measurements were performed using hook-shaped stainless steel needle [14]. For the contact angle measurements, the testing liquid volume was 3 μL with an equilibration time of 3 s.

### 2.5. Membrane Permeation Tests

**Figure 1:**
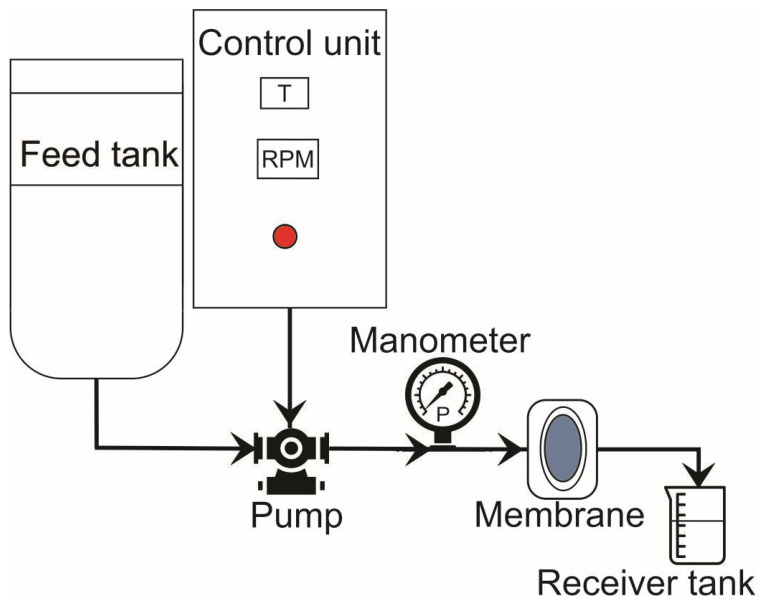
Dead-end filtration setup for membrane performance tests.

Hydrodynamic water flux and emulsion flux were measured on the dead-end filtration setup illustrated in Figure 1. In the separate experiments, pure water and oil-in-water emulsions were poured into the feed tank and passed through the membrane at pressures from 0.5 to 2.5 bar. Permeate was collected, and oil droplets particle size distribution was analyzed using DLS particle size analyzer (Anton Paar, Graz, Austria).

The permeate flux values were calculated according to Equation (1):

(1) $$J_{v}=\frac{\Delta m}{A\Delta t},$$

where J_v_ is the permeate membrane flux, (kg m^−2^ h^−1^); Δm is the mass of permeate (kg) passed through the membrane area A (m^2^), over the time of the experiment Δt (h).

## 3. Results and Discussion

**Figure 2:**
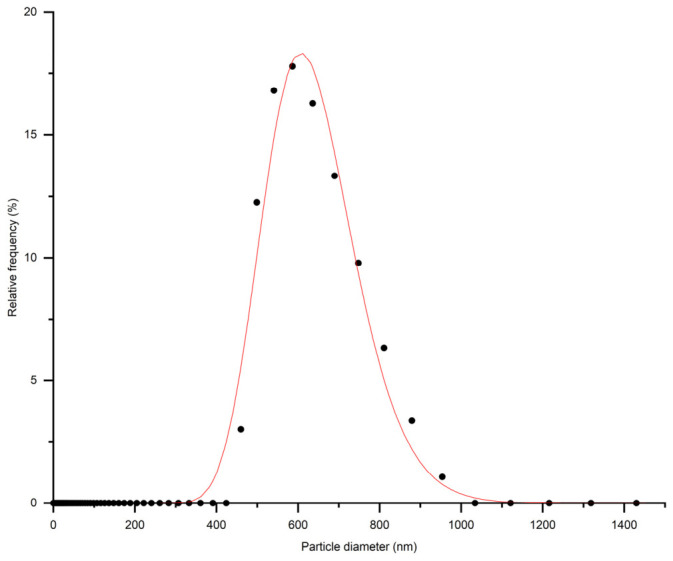
Particle size distribution of CFA suspension.

Particle size analysis of the CFA suspension in water with concentration of 5·10^−4^ g dm^−3^ showed that the average ash particle size was close to 620 ± 12 nm (Figure 2). In the analyses, only results exhibiting a polydispersity index below 40% were selected.

Zeta potential of CFA suspension in water was measured to comprehend the CFA’s surface properties. The measured zeta potential was equal to −25 mV and this result can be explained by the presence of aluminum and silicon oxides in CFA particles carrying the negative charge on the surface when mixed with water, which has also been confirmed in previous studies [15]. Also, this value of the zeta potential implies the low stability of CFA particles and the tendency to aggregate over time, explained by weak repulsive forces between negatively charged particles of ash overcome by stronger van der Waals forces [16,17]. To obtain a smooth and monodisperse layer of fly ash on the TiO_2_ membrane surface, a stable suspension of CFA was needed. To prevent the aggregation of dispersed CFA particles, stabilization with cationic surfactant was performed. Cationic surfactants were chosen because of the negative CFA surface charge. CFA particles tend to aggregate owing to the low negative charge of the surface; therefore, an addition of the cationic surfactant can neutralize and then reverse the charged surface at certain concentrations. The stabilization of CFA suspensions in the presence of cationic surfactants is likely governed by electrostatic interactions between negatively charged CFA particles and positively charged surfactant molecules, as described in Derjaguin, Landau, Verwey, and Overbeek theory (DLVO) [18]. Although zeta potential measurements of surfactant-modified suspensions were not performed, this interpretation is supported by the observed changes in particle size distribution. The influence of N-dodecylpyridinium chloride (DPC), (1-hexadecyl)trimethylammonium bromide (HDTA), and their combination effect on the CFA suspension stability was investigated.

**Figure 3:**
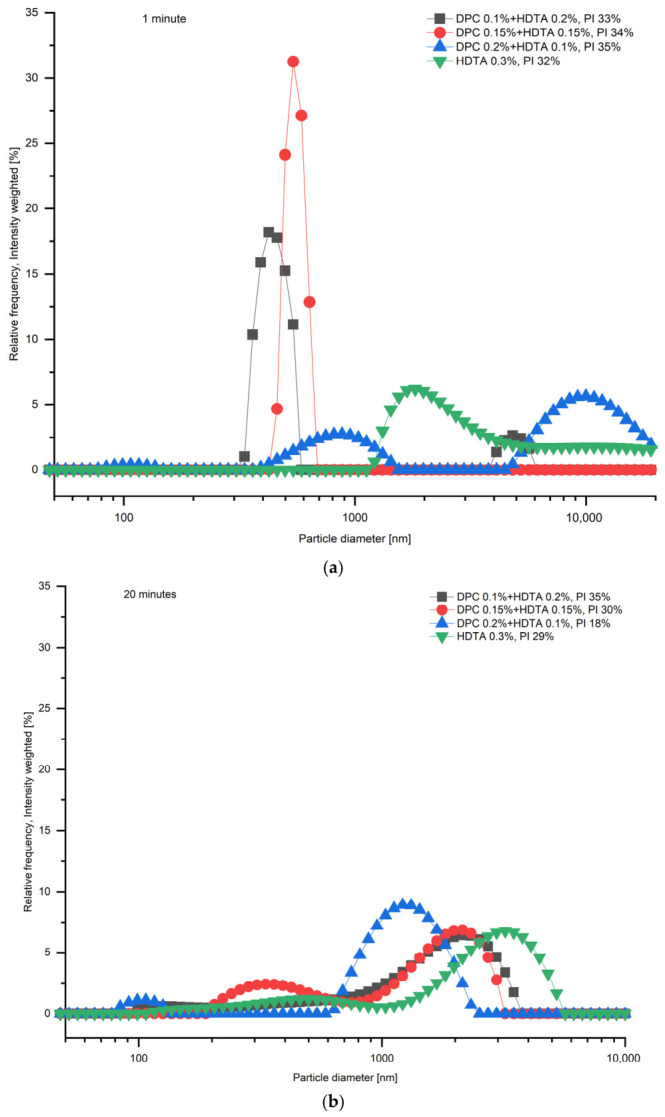
Particle size distribution of CFA suspensions containing DPC and HDTA measured at (**a**) 1 min and (**b**) 20 min after suspension preparation. PI—polydispersity index.

According to the particle size distribution analysis, the addition of HDTA and combination with DPC was not effective in terms of ash particle stability. Figure 3 illustrates that the severe agglomeration of CFA particles takes place after obtaining the CFA suspension in water and the size of particles increased to more than 1000 nm.

**Figure 4:**
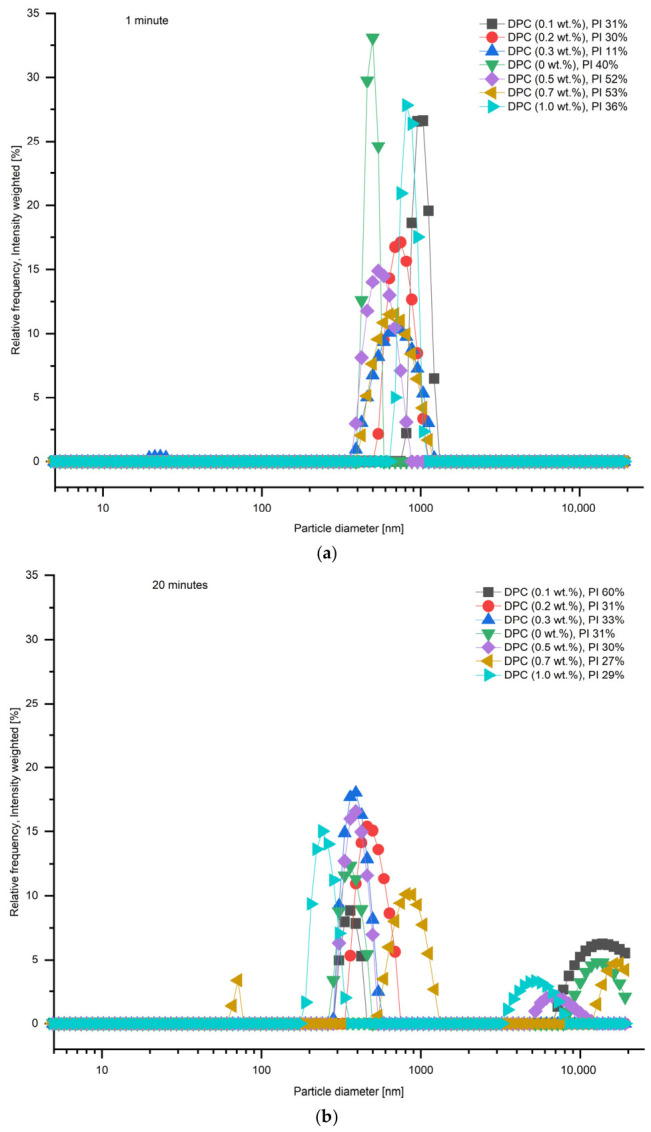
Particle size distribution of CFA suspensions with different DPC concentrations measured at (**a**) 1 min and (**b**) 20 min after preparation. PI—polydispersity index.

Furthermore, the effect of DPC concentration was investigated with respect to the CFA suspension stability. Figure 4 demonstrates that the stability of CFA particles in suspension decreases over time, and the smallest CFA particles can be obtained during the first 10 min after the suspension preparation. An addition of DPC to the CFA suspension does not retard agglomeration significantly; therefore, the optimal concentration of 1.0 wt% was chosen for further investigations.

According to the WCA measurements (Table 2, Figure S1), it is clear that the modified surface became much more hydrophilic owing to the reduced WCA (the change from 43.5° to around 0°) and under oil contact angle (UOCA) values for water droplets (from 147.6° to 87°) with cyclohexane as the oil phase. In contrast, underwater contact angle (UWCA) measurements for cyclohexane droplets demonstrate that the surface turned more oleophilic with a decrease in UWCA_cyclohexane_ values from 112.1° to 101.3° while CA values for cyclohexane in air has not changed significantly. This can be related also to the altered surface roughness that also impacts CA values [19].

**Figure 5:**
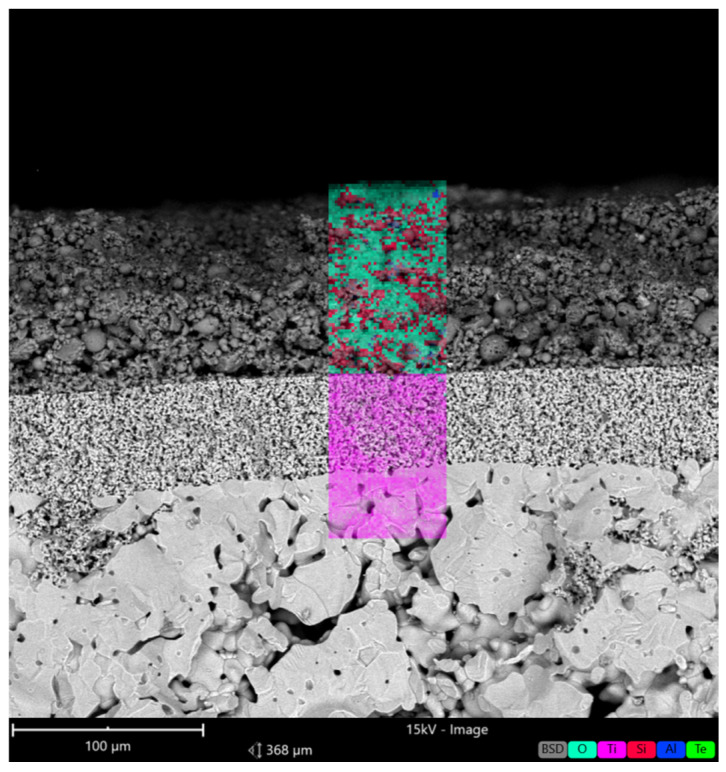
SEM-EDX image of the modified membrane.

To justify the pristine TiO_2_ membrane surface modification, SEM-EDX images were obtained and results are presented in Figure 5.

According to the SEM-EDS image obtained (Figure 5), it is clear that there is a layer of CFA particles on the separation layer and the elemental composition (Table 3) shows the presence of silicon, aluminum, and oxygen.

Zeta potential values of pristine and modified membranes at different pH values were measured to understand how membrane surface changes after coating with CFA layer.

**Figure 6:**
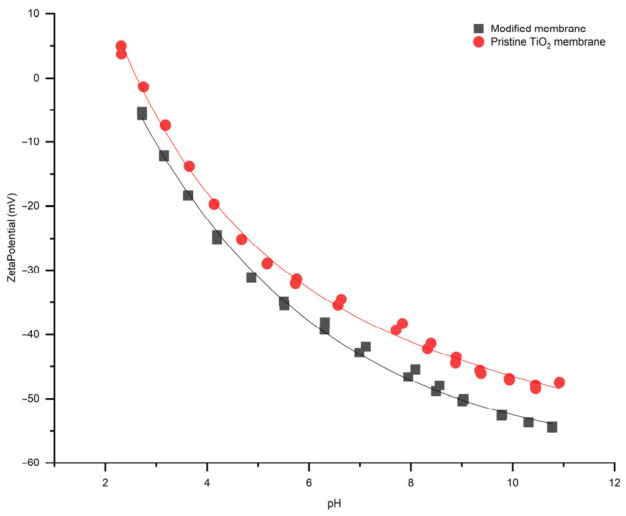
Zeta potential of the modified and pristine membrane with respect to pH.

According to the zeta potential measurements (Figure 6), the surface charge decreased slightly after surface modification with the difference in 4.5 mV at pH 2.7. The surface charge of the pristine and modified membranes correlates with the existing data [20,21], and the zeta potential becomes increasingly negative as the pH increases for both membranes, indicating higher surface charge at higher pH values. At lower pH values, the zeta potential is closer to zero or even slightly positive, especially for the pristine TiO_2_ membrane. The modified membrane exhibits a consistently more negative zeta potential than the pristine membrane across the entire pH range. The modification process introduces functional groups, e.g., hydroxide groups, that increase the negative surface charge density of the membrane, as evident from the more negative zeta potential values. Moreover, it should be noted that changes in zeta potential may be attributed to variations in surface roughness and pore size [22,23]. This difference in zeta potential affects membrane properties like antifouling performance, selectivity, and filtration efficiency. In particular, a more negative zeta potential enhances antifouling properties due to increased electrostatic repulsion between the membrane surface and negatively charged foulants, thereby reducing their adsorption and deposition on the membrane surface. The modified membrane, exhibiting a greater negative zeta potential, is suitable for applications demanding stability at greater pH levels.

**Figure 7:**
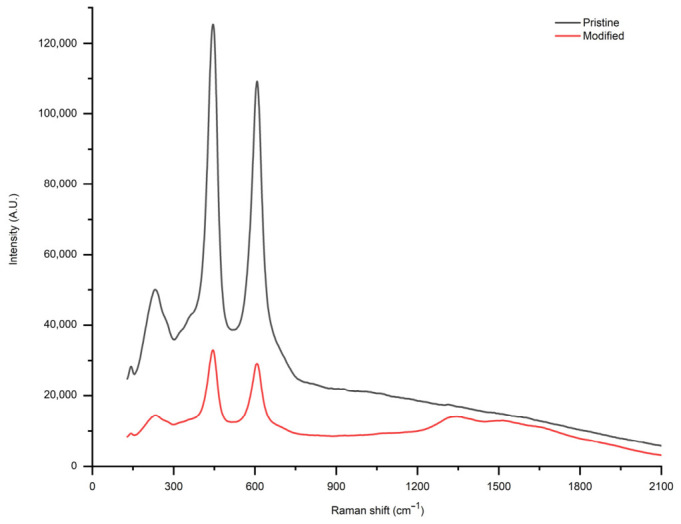
Raman spectrum of pristine and modified membranes.

Raman spectrum of the pristine membrane presented in Figure 7 correlates well in positions of shifts with other studies [24,25], with the most intensive peaks at 446, 608.5 and 233.5 cm^−1^. The pristine TiO_2_ membrane shows strong Raman signals at its characteristic peaks, confirming the presence of TiO_2_. In contrast, the modified membrane exhibits lower peak intensity, which is attributed to the partial coverage of the TiO_2_ surface by fly ash components. The dominant peaks for pristine TiO_2_ appear in the low-wavenumber region (200–800 cm^−1^), characteristic of the rutile phase. According to the Raman spectrum of the modified membrane, there were no characteristic bands of mullite, Al_2_O_3_, and SiO_2_ as reported elsewhere [26,27]. The absence of characteristic Raman peaks of CFA components may be due to the dominant signal of the rutile TiO_2_ phase, which can mask weaker contributions from CFA. In addition, the deposited CFA layer is relatively thin, and fly ash may contain amorphous phases that produce weak or indistinct Raman features.

**Figure 8:**
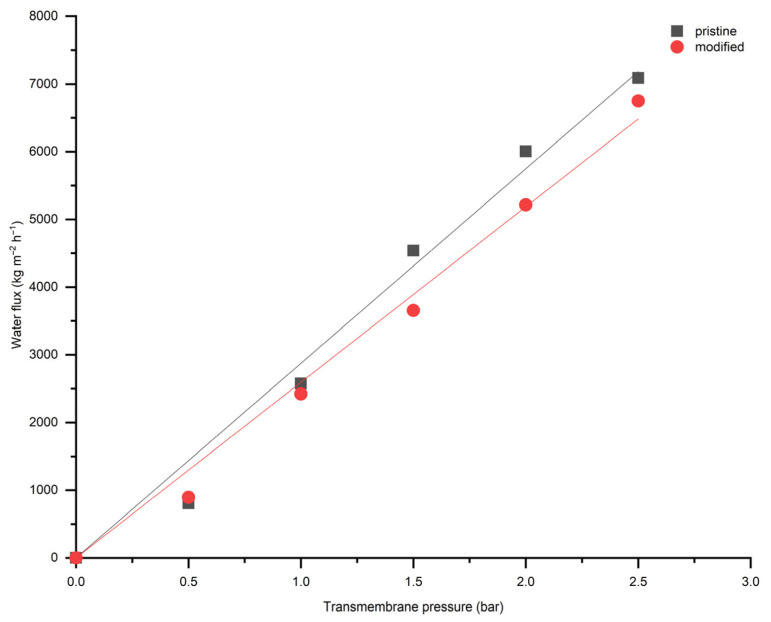
Water flux as a function of transmembrane pressure for TiO_2_ and modified membranes.

Water flux measurements were performed for both pristine TiO_2_ and modified membranes (Figure 8). According to the results in Figure 8, membrane modification did not significantly affect water permeation through the modified membrane significantly and showed a linear relation with correlation coefficient (R^2^) above 0.99. The linear correlation with increasing transmembrane pressure indicates pressure-driving forces flow through a stable porous structure after modification without compaction or pore blockage within the investigated pressure range.

**Figure 9:**
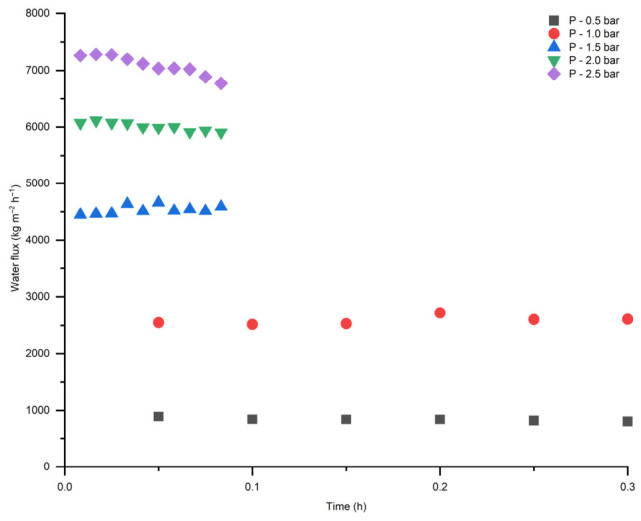
Water flux as a function of time for pristine TiO_2_ membrane.

**Figure 10:**
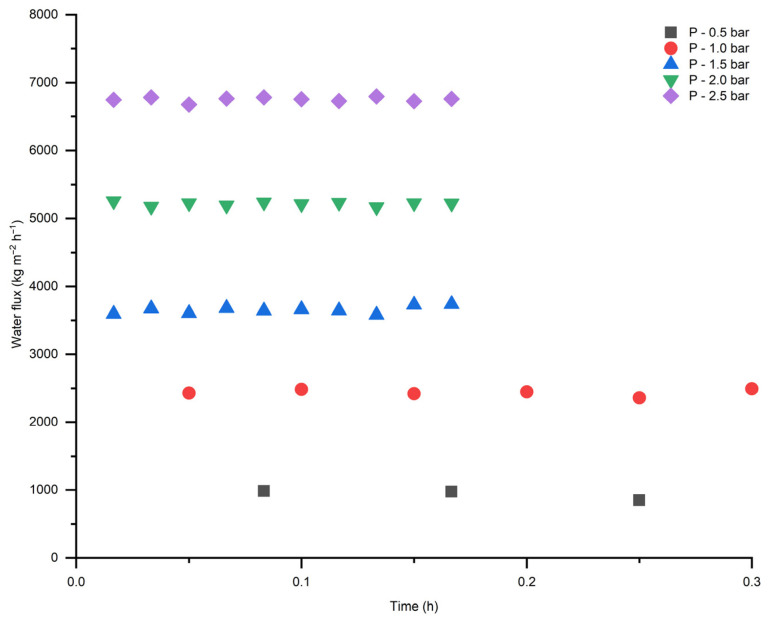
Water flux as a function of time for modified TiO_2_ membrane.

Both modified and pristine membranes demonstrated stable water flux at different pressures (from 0.5 to 2.5 bar) (Figure 9 and Figure 10). This is related to the fact that the ceramic membranes are more robust in terms of mechanical strength and there is no swelling compared to the polymeric membranes [28]. Despite the enhanced hydrophilicity, the modified membrane exhibited a slight decrease in water flux compared to the pristine membrane, which can be attributed to partial pore blockage and the formation of a surface layer, as evidenced by the SEM-EDX image (Figure 5).

**Figure 11:**
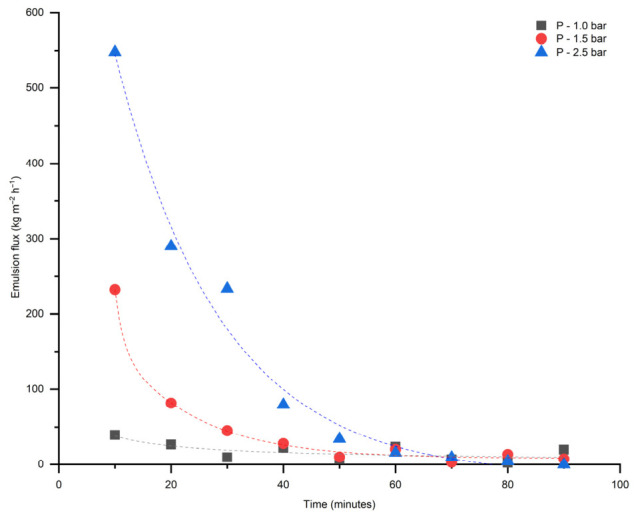
Oil-in-water emulsion flux as a function of time for pristine TiO_2_ membrane.

**Figure 12:**
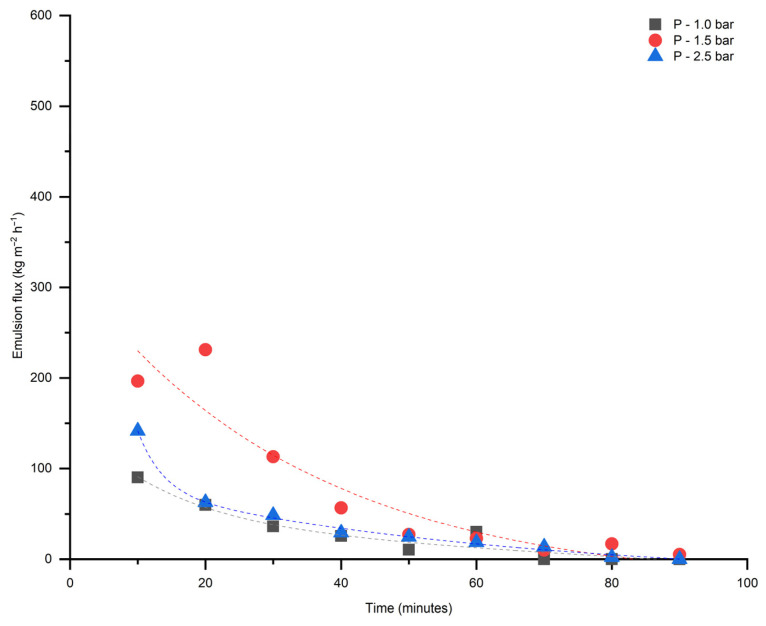
Oil-in-water emulsion flux as a function of time for modified TiO_2_ membrane.

The fluxes of 5 vol% of heptol-in-water emulsion stabilized by Tween 80 through pristine and modified membranes were measured and results are presented in Figure 11 and Figure 12.

The modified membrane has shown improved wettability, but suppressed pure water and oil-in-water emulsion flux. This improvement is evidenced by a significant decrease in the water contact angle from 43.6 ± 2° to approximately 0°, indicating enhanced hydrophilicity. In addition, the underoil contact angle of water decreased from 147.6 ± 2° to 87 ± 2°, implying that the membrane surface became more hydrophilic even in an oil medium (Table 2). Additionally, once membrane surface becomes hydrophilic, further decreasing in-contact angle does not noticeably enhance water transport, as the dominant resistance arises from flowing through the membrane pores rather than surface wetting effects. Moreover, these findings show that the fly ash layer increases the membrane affinity toward water both in air and under oil, causing better wettability and potentially reducing fouling by oily species.

These changes in interfacial behavior reflect the influence of fly ash incorporation on the surface chemistry and energy of the TiO_2_ membrane. Nevertheless, oil-in-water emulsions passing through the modified membrane gained greater stability and smaller oil droplet sizes.

**Figure 13:**
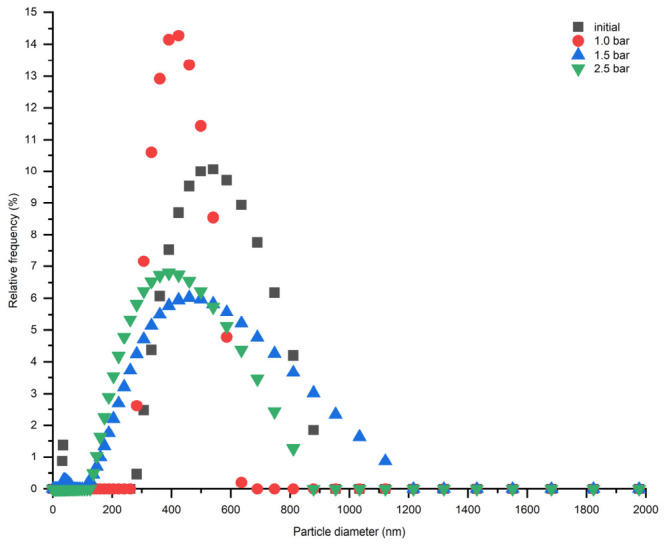
Particle size distribution of the oil droplets in 5% o/w emulsion and after passing it through the pristine membrane in the first 10 min.

**Figure 14:**
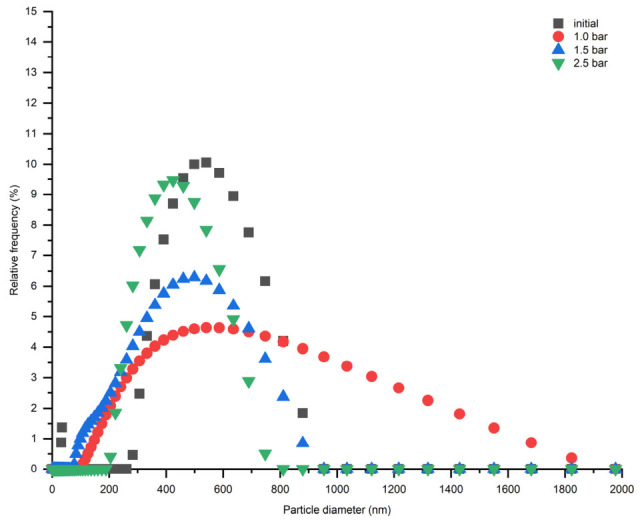
Particle size distribution of the oil droplets in 5% o/w emulsion and after passing it through the modified membrane within the first 10 min.

There is no significant difference in the particle size distribution of the oil droplets in the o/w emulsions after passing them through the membranes (Figure 13 and Figure 14). It should be noted that oil droplets become smaller during the membrane test (at the highest polydispersity index (PI) values) for both pristine and modified membranes.

To achieve charge and steric stabilization of CFA suspension for TiO_2_ membrane modification, 1.0 wt% of DPC solution was chosen. HDTA addition to the CFA suspension did not result in high stability of ash particles. This limited effect may be attributed to steric hindrance around the nitrogen atom in the HDTA molecule, which could reduce its ability to interact with the negatively charged surface of the ash particles. In contrast, stabilization by DPC molecules with spatially available nitrogen atoms carrying a positive charge can form a layer of molecules on the CFA particles. Also, PVA solution contributes to steric stabilization, preventing CFA particles from agglomeration. It should be noted that PVA mainly acts as a temporary binder during coating and is expected to decompose during sintering; therefore, it does not directly affect the final surface chemistry of the modified membrane, although its influence on the microstructure of the coating layer cannot be entirely excluded.

The Raman analysis indicates significant changes in the chemical properties of the membrane post-modification. The pristine TiO_2_ membrane displays characteristic peaks of the rutile phase. After modification with fly ash, slight changes in the Raman spectrum suggest that fly ash components partially cover the TiO_2_ surface or interact with it, leading to subtle alterations in the observed spectral features. Despite the reduced intensity, no additional peaks were detected in the modified membrane spectrum, which suggests that the modification does not introduce significant new crystalline phases, such as mullite or other alumina–silica-based components typically found in fly ash.

The zeta potential measurements provide additional insights into the surface properties. The modified membrane exhibits a more negative zeta potential across all pH levels compared to the pristine membrane. This indicates the successful introduction of hydroxide groups or a surface layer from fly ash that enhances the negative surface charge. This increased negative charge is beneficial for antifouling properties and stability under alkaline conditions, as it promotes electrostatic repulsion between membrane and foulants, thereby minimizing fouling and improving membrane performance [29,30].

In terms of hydrophilicity, the modified membrane displays a considerable decrease in water contact angle from 43.5 ± 2° to nearly 0°, which highlights an improvement in wettability (Table 2). Such a surface is advantageous for water treatment applications, particularly in emulsified oil separation, as it promotes the spread of water over the surface, reducing fouling and enhancing filtration efficiency.

The water flux experiments further validate the mechanical robustness and hydrophilic properties of the modified membrane. The linear relationship between water flux and transmembrane pressure indicates that the structural integrity remains intact post-modification. The oil-in-water emulsion filtration results, demonstrating smaller oil droplet sizes post-filtration, suggest enhanced emulsification stability attributed to the modified surface properties.

The dead-end configuration of the experimental setup was found to be ineffective in breaking emulsions. Alternative setups, including cross-flow and stirred-tank systems, may provide improved performance and should be systematically evaluated in future studies.

## 4. Conclusions

The modification of TiO_2_ ceramic membranes with coal fly ash (CFA) successfully enhanced their surface and interfacial characteristics without compromising structural integrity or water permeability. The applied slip-casting approach enabled the uniform deposition of a CFA-derived layer, as confirmed by SEM-EDX, which revealed the presence of silicon- and aluminum-rich oxides on the TiO_2_ surface. This compositional modification significantly influenced the membrane’s surface chemistry, leading to improved hydrophilicity and altered electrokinetic behavior.

Contact angle measurements demonstrated that the modified membrane became superhydrophilic, with the water contact angle decreasing from 43.5 ± 2° to nearly 0°, and the underoil contact angle of water reducing from 147.6 ± 2° to 87 ± 2°. These results indicate that the fly ash layer enhanced the membrane affinity toward water both in air and under oil, promoting better wettability and potentially reducing fouling by oily species. The zeta potential measurements further confirmed the introduction of additional negatively charged functional groups, which increased the negative charge of membrane surface across the entire pH range. Such behavior is advantageous for stability under alkaline conditions and improves resistance to organic or colloidal fouling through electrostatic repulsion effects.

Raman spectroscopy confirmed the retention of TiO_2_ spectral features while also revealing subtle changes associated with chemical interaction between TiO_2_ and CFA components, supporting the successful formation of a composite surface layer. The modified membrane maintained stable and linear water flux with increasing pressure, demonstrating that the modification process did not impair mechanical robustness or permeability. Although the filtrate analysis revealed a slight decrease in apparent oil droplet size after passage through the modified membrane, this may suggest a potential increase in emulsion stability. However, no direct stability measurements (e.g., turbidity evolution or zeta potential of the emulsion droplets) were conducted.

Overall, this study demonstrates that coal fly ash can be effectively repurposed as a low-cost and sustainable material for modifying TiO_2_ ceramic membranes. The approach offers dual environmental benefits: valorization of industrial waste and enhancement of membrane performance in oil–water separation applications. The improved hydrophilicity, negative surface charge, and stable filtration behavior make CFA-modified TiO_2_ membranes a promising option for use in wastewater purification, emulsified oil treatment, and related separation processes, providing an environmentally friendly route toward high-performance ceramic membrane materials. However, further studies are required to evaluate the long-term stability and reusability of the modified membranes through repeated cycling tests.

## Figures and Tables

**Table 1:** Casting solution composition.

Component	Mass [g]	Weight Fraction [wt%]
CFA	2.94	3.92
PVA	2.65	3.50
DPC	0.96	1.28
Deionized water	68.45	91.30

**Table 2:** Contact angle measurements.

	Pristine TiO_2_ Membrane	Modified TiO_2_ Membrane
CA_water_ [°]	43.5 ± 2	0.0 ± 2
CA_cyclohexane_ [°]	7.0 ± 2	10.7 ± 2
UWCA_air_ [°]	113.5 ± 2	112.0 ± 2
UWCA_cyclohexane_ [°]	112.1 ± 2	101.3 ± 2
UOCA_water_ [°] ^1^	147.6 ± 2	87.0 ± 2

^1^ the heavy phase is written as a lower index.

**Table 3:** Elemental composition of membrane selected area.

Element	Average Weight Fraction [wt%]
Oxygen	60.0
Titanium	19.7
Silicon	12.6

## Data Availability

The original contributions presented in the study are included in the article. Further inquiries can be directed to the corresponding author.

## Supplementary Materials

The following supporting information can be downloaded at: https://www.mdpi.com/article/10.3390/membranes16050157/s1, Figure S1: Contact angle images.

## Abbreviations

The following abbreviations are used in this manuscript:

A	Membrane area [m^2^]
CFA	Coal fly ash
DLS	Dynamic light scattering
DPC	1-Dodecylpyridinium chloride
HDTA	(1-Hexadecyl)trimethylammonium bromide
HLB	Hydrophilic–lipophilic balance
J_v_	Permeate membrane flux [kg m^−2^ h^−1^]
PL	Polydispersity index
PVA	Polyvinyl alcohol
SEM-EDX	Scanning electron microscopy with energy-dispersive X-ray
TiO_2_	Titanium oxide
TPP	Thermal power plant
UOCA	Under oil contact angle of water [°]
UWCA	Under water contact angle [°]
WCA	Water contact angle [°]
