# Supplementary material for: Ceramic TiO2 Membrane Modification by Coal Fly Ash (CFA) Particles
Source: Membranes (Basel). 2026 Apr 29;16(5):157. doi: 10.3390/membranes16050157 (PMC13209068; doi:10.3390/membranes16050157)
Supplement: Supplementary file 1 [file membranes-16-00157-s001.zip › membranes-4225057-supplementary.pdf]

## Supplementary materials

| | Pristine $TiO_2$ membrane | Modified $TiO_2$ membrane |
|---|---|---|
| $CA_{water}$ [°] | 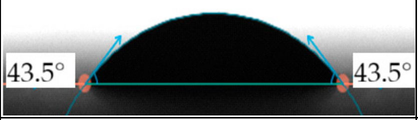 | 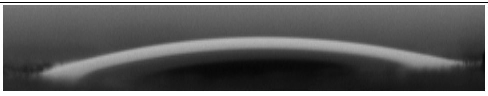 |
| $UWCA_{cyclohexane}$ [°] | 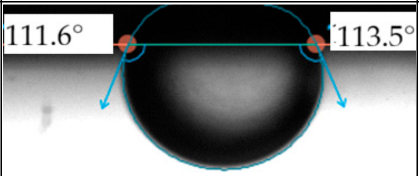 | 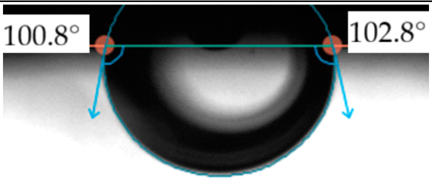 |
| $UOCA_{water}$ [°] | 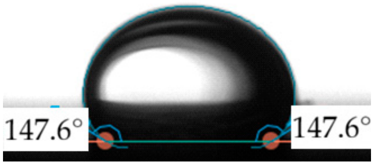 | 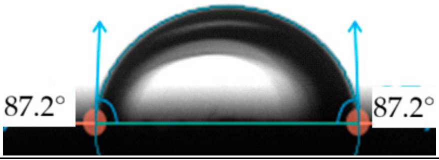 |

CA – contact angle, UWCA – underwater contact angle, UOCA – underoil contact angle

**Figure S1.** Contact angle images.

**Disclaimer/Publisher's Note:** The statements, opinions and data contained in all publications are solely those of the individual author(s) and contributor(s) and not of MDPI and/or the editor(s). MDPI and/or the editor(s) disclaim responsibility for any injury to people or property resulting from any ideas, methods, instructions or products referred to in the content.

https://doi.org/10.3390/membranes16050157
